# Family-Based Intervention Program for Parents of Substance-Abusing Youth and Adolescents

**DOI:** 10.1155/2016/4320720

**Published:** 2016-10-05

**Authors:** David Bisetto Pons, Remedios González Barrón, Álvaro Botella Guijarro

**Affiliations:** ^1^Faculty of Psychology, University of Valencia, Valencia, Spain; ^2^AEPA Foundation, Alicante, Spain

## Abstract

The use of drugs among adolescents/youth often results in a high degree of distress for the family members who live with them. This in turn can lead to a deterioration of mental (psychological) health, hindering any attempt to successfully cope with the situation. The goal of our research was to study the effect of the Community Reinforcement and Family Training (CRAFT) program on parents of adolescents/young adult drug users. Study volunteers (*n* = 50) were parents from Valencia (Spain) that were divided into two groups. The experimental group (*n* = 25) was made up of parents whose sons and daughters exhibited problems with drug use and the constructed noncausal baseline group (*n* = 25) was made up of parents whose sons and daughters did not show any substance abuse problems. For both groups, self-esteem (Rosenberg Self-Esteem Scale), depression (BDI-II), anxiety (STAI), and anger (STAXI-II) were evaluated before and after the application of the CRAFT program. Results show a significant improvement in the experimental group's self-esteem, depression, and anger state and a decrease in negative moods. These changes in parents produce a positive effect on their substance-using sons and daughters: of the 25 participants, 15 contacted specialized addiction treatment resources for the first time.

## 1. Introduction

There are a considerable number of studies which suggest that parents and other relatives of adolescent/young adult substance users experience a high degree of distress and family conflict that could result in a deterioration of mental health [1, 2]. The negative impact on the family of adolescents/youth with substance use problems is comparable to the impact on people living with an adult with the same problem [2]. It is estimated that approximately five people close to the adult substance abuser will be directly affected by their addiction. This figure also holds true for families of adolescent/young adult substance user [3]. The adverse effects experienced by the parents of substance users take the form of physical, mental, and social stress which can lead to depression, somatic ailments, low self-esteem, a high degree of anxiety and anger, fear that their son/daughter is in danger, despair, guilt, and pain arising from the feeling that they have failed as parents [2, 4–6]. In the words of Orford et al. [4], affected family members are “*ordinary people trying to cope with highly stressful experiences*.” All of these manifestations are similar to those experienced during prolonged periods of stress or adversity, such as war, long-term unemployment, one's own chronic illness, or the illness of a family member living in the same household [5].

Furthermore, the importance of family support during the recovery process of a person with a substance abuse disorder has been widely shown. For instance, Casas and Gossop [7] suggest that family pressure has an influence on the user's decision to stop using alcohol or other drugs (hereinafter AOD). Levy [8], in his five-year follow-up study of narcotic addicts, found that subjects who overcame addiction usually did so because family support was part of the process.

Booth et al. [9] found that family support received by substance users during addiction treatment led to increased self-esteem and personal efficacy and led them to remain in treatment as a result. López-Torrecilla et al. [10] indicate a greater degree of personal efficacy among substance users whose relatives were involved in their addiction treatment.

Similarly, for adolescent/young adult substance users, a number of authors have observed that family support favors the processes of detection, prevention of problematic drug use, and probability of initiating and remaining in treatment [11–14]. Improvement has also been observed in individual treatment programs, leading to fewer relapses, improved family relations, and a higher probability of reducing the use of AOD among substance users [15]; these substance users are also more likely to distance themselves from settings and relationships associated with AOD abuse behaviors [16, 17], helping them put an end to drug abuse [18].

The following data is a summary of the situation in Spain regarding the use of AOD among adolescents/youth: adolescents start using drugs between the ages of 13 and 16 for most substances. Alcohol, tobacco, and cannabis continue to be the most frequently used drugs among Spanish adolescents/youth. It was estimated that 52.2% of youth aged between 15 and 24 years smoke cannabis [19]. In addition, cannabis use is the reason for 93% of all requests for treatment among adolescents between 14 and 18 years of age; this indicates a growing trend in the problematic or high-risk use of cannabis, which is associated with lower academic performance [19, 20].

As a developmental stage, adolescence/youth is marked by significant psychological, physiological, and social changes [21]. For this reason, it is considered a stage where the young adult is most at risk, favoring the appearance of mental disorders such as anxiety, impulsive and aggressive behavior, stress, and depression [22–24], which are associated with a greater degree of family conflict [25]. This is coupled with an observed desire to experiment with substances while playing down the danger they pose, overconfidence, and a false sense of being in control. All of these factors increase the risk of suffering from problems associated with the use/abuse of AOD [26] and increase the chance of developing drug dependency problems in the future [27–29] which may not be seen by the substance abuser as being problematic [30].

In summary, studies indicate the existence of an adolescent/young adult population with substance use problems. This risky use of AOD and the circumstances that often surround this behavior have been shown to affect the mental health of their families, among other reasons, because this situation exposes family members to a high degree of stress. When they appear, these effects on the mental health of other members of the family make it more difficult for the same family members to act appropriately when it comes to detecting and preventing substance use and also prevent them from initiating and remaining in an addiction treatment program. In light of these considerations, effective addiction treatment should include support for family members, empowering them to take control of the situation wherever they can.

There are a number of empirically validated intervention programs which work towards improving mental distress experienced by close relatives of adolescents/youth who are substance abusers [1]; these include the 5-Step Method [31], the Adolescent Transition Program (ATP) [32],* BEST*, and* BEST-Plus* [33, 34].

The Community Reinforcement and Family Training (CRAFT) program developed by Smith and Meyers [35] has been found to be effective in both adults resistant to starting treatment [36] and adolescent/young adult substance abusers [37]. This treatment program has been found to improve the mental health of non-substance-abusing family members by encouraging the adolescent to cease using said substances [38, 39]. The CRAFT program is recommended when working with non-substance-abusing family members because it increases their self-esteem, improves symptoms associated with depression and anxiety, and reduces distress and anger [36, 40].

The CRAFT program [35] is divided into two large sections. The first provides training in behavior modification techniques. This section consists of the following components: strategies that encourage participants to actively engage in treatment, identification of high-risk situations through a functional analysis of the family member's drug-using behavior, prevention of domestic violence, training in positive communication skills, identification of activities which reinforce positive behavior and compete with drug use, training on how to remove reinforcement of substance-abusing behavior, assistance to family members in planning lifestyle changes in those areas of their lives which they feel are unsatisfactory, problem solving, and training that helps them encourage adolescents with drug problems to enter addiction treatment program or seek out specialized assistance.

The second portion of the program focuses on the adolescent/young adult once they have entered addiction treatment program with the support of their family.

In this study, the program was adapted for use on a Spanish group of participants. It was implemented using the group therapy approach developed by Foote and Manuel [41].

The objective of this pilot study was to analyse, in the Spanish population, how the first part of the CRAFT program helps parents of adolescent/young adult substance users acquire or use skills that allow them to improve their mental well-being and therefore assist their children in initiating an addiction treatment program [42].

To this end, the following working hypotheses have been established.

- There are statistically significant differences in self-esteem, depression, state anxiety and trait anxiety, state anger, trait anger, and anger expression index (AX Index) scores between the group comprising family members of adolescent/young adult substance users before treatment and the group of family members who state that they do not have any problems (the noncausal baseline group).
- The family members targeted by the intervention will show improved scores in the variables mentioned above, obtained after treatment, when compared to scores obtained prior to treatment.
- Scores obtained after treatment for study variables will not differ significantly from those obtained in the constructed noncausal baseline group.
- At least 50% of the members of the experimental group will help their substance-abusing relative to seek out help from a specialized resource for the treatment and indicate prevention of substance abuse (Addictive Behavior Unit and Community Prevention Unit) before treatment program completion (ten weeks).

## 2. Materials and Methods

### 2.1. Research Classification

The study is a quantitative, pretreatment, and posttreatment study with a constructed noncausal baseline (hereafter CNCB) [43, 44]. The “baseline” here refers to the scores obtained in the study variables by the parents who did not have adolescent/youth with AOD use problems.

### 2.2. Participants

To carry out the study, the authors performed convenience sampling. The parents of adolescent/young adult substance users were referred from the Addictive Behavior Unit (Spanish acronym: UCA), Community Prevention Unit (Spanish acronym: UPCCA), and the Social Services Department. These resources were contacted to offer the intervention program for parents with adolescent/young adult substance users. None of the participants had a family history of addiction.

All participants stated that none of the siblings of substance users had experienced problems associated with drug use.

All of the parents in the first group claim to have or have had mental, social, and even somatic problems and a family atmosphere they classify as being either “*quite bad*” or “*very bad*.”

At the same time, convenience sampling was performed to select participants for the CNCB group. These were parents whose sons or daughters did not have any substance use problems and were not suffering from any other stressful life situations that could give rise to similar symptoms of distress. This information was provided by the parents themselves.

### 2.3. Instruments

Sociodemographic characteristics were collected by way of a semistructured interview conducted with each of the participants, adapted from Cortés-Tomás and Pascual-Pastor [45].

Self-esteem was evaluated through* Rosenberg's Self-Esteem Scale* [46], adapted by Echeburúa [47], and consisting of ten items answered on a four-point scale (1: strongly agree, 2: agree, 3: disagree, and 4: strongly disagree). The reliability and validity of this instrument are 0.87 and 0.72, respectively [48].

To evaluate depression, the* Beck Depression Inventory* (BDI-II) was used [49]. This inventory is composed of 21 items, each with four possible responses that are assigned a score indicating the severity of the symptom (arranged in order from mild to severe). It has a high internal consistency, in both clinical and nonclinical samples, with an alpha score of 0.92. The Spanish version of this test also shows high internal consistency both in samples with university students (0.80) and in general or clinical population [50].

Anxiety was evaluated using the* State-Trait Anxiety Inventory* (STAI) [51], composed of two Likert-type scales with a total of 40 items that measure state anxiety (A-State) and trait anxiety (A-Trait). As far as reliability and validity are concerned, this inventory has been tested on the Spanish population and shows an internal consistency of between 0.90 and 0.93 for state anxiety and between 0.84 and 0.87 for trait anxiety.

Anger was evaluated through the State-Trait Anger Expression Inventory (STAXI-2) [52]. The items that it contains measure state, trait, and expression of anger [53]. The inventory shows good psychometric properties that are internally consistent with an alpha score of 0.89 for the State Anger Scale and 0.82 for the Trait Anger Scale.

### 2.4. Procedure

Once the participants of the experimental group were referred by the various collaborating bodies, they were evaluated by a psychologist using the instruments described above.

The experimental group set to receive the adapted CRAFT program was divided into four smaller intervention groups [35], consisting of six participants each, except for one group, which had seven participants. Each intervention consisted of ten weekly sessions lasting one hour and thirty minutes each. The sessions were given by the same therapists.

### 2.5. Data Analysis

The percentage of families with problems associated with AOD use that sought out some kind of addiction treatment or intervention at some time during the study was calculated based on the information provided by the parents attending the group sessions.

An exploratory analysis was conducted to identify possible outliers and missing data and determine data distribution, symmetry, kurtosis, homoscedasticity, and other aspects. These analyses revealed that, given the study samples, it could not be stated that the data are from a normally distributed population for the variables under study (Shapiro-Wilk test). Furthermore, outlying scores were found for all variables, and the variances of the groups to be compared were found to be equal (Levene's test to assess homogeneity of variance).

In light of the above findings, the authors decided to use nonparametric tests for inferential analyses. While these tests offer statistical robustness, they have less power; that is, their *p* values tend to be higher and there is a greater chance of type II errors.

Scores obtained before treatment were compared to scores taken after treatment in the experimental group, using the Wilcoxon signed-rank test, the nonparametric version of the *t*-test for dependent samples.

Scores at pretreatment for each variable were compared to scores from the constructed noncausal baseline group to see if there were differences in the study variables between families of adolescent/young adult substance abusers before intervention and families that stated that they did not have any drug-related problems. To make this comparison, the Mann–Whitney *U* test was used, which is the nonparametric equivalent to the *t*-test for independent samples.

Lastly, the scores taken from the experimental group after treatment were compared to those of the noncausal baseline group to determine whether any differences observed before treatment between the substance-user family member group and the CNCB group had diminished. The Mann–Whitney *U* test was also employed for this analysis.

Effect sizes were calculated for each comparison, using the formula $r=Z/\sqrt{N}$, where *r* is the effect size, *Z* is the *U* or *W* score, depending on the test employed, converted to a *Z* score, and *N* is the total size of the sample, that is, the sum of both sample sizes of each group (50 in this case). The coefficient of determination (*r* ^2^) was calculated to allow for a better interpretation of the effect size according to the proportion of explained variance.

To conduct this statistical analysis, the R computing software environment was used, version 3.2.3 Wooden Christmas-Tree, released 10-12-2015, using the coin package [54], with the* RStudio* integrated development environment, version 0.99.491.

## 3. Results

A summary of sociodemographic variables is provided in Table 1. No statistically significant differences were observed between the experimental and constructed noncausal baseline groups in terms of parental age (*t*(48) = −1.80; *p* = 0.078, two-tailed) or gender (*x* ^2^  (1, *N* = 50) = 0.397; *p* = 0.754, two-tailed).

*Hypothesis a*. The results obtained before treatment by the experimental group, when compared to those obtained from the CNCB group, show statistically significant differences (*p* < 0.01) for all variables except state anxiety and anger expression index (AX Index) (Table 2).

In particular significant differences were found in self-esteem (*p* < 0.01), where the means were found to be higher for the constructed noncausal baseline group. The experimental group showed higher means for depression (*p* < 0.01), trait anxiety (*p* < 0.01), and state anger and trait anger (*p* < 0.01). The effect sizes (*r*) indicate a correlation between the particular group each member belongs to and each of the variables, in terms of absolute values. For instance, for the self-esteem variable, a correlation of 0.65 was found between membership to one of the groups and self-esteem scores. The determination coefficient (*r* ^2^) indicates the percentage of variance which predicts membership to one or the other group. For the self-esteem variable, this was found to be 42.2%.

*Hypothesis b*. Pretreatment and posttreatment results were compared for the experimental group (Table 3). All of the variables under study improved, with statistically significant differences obtained for self-esteem (*p* < 0.01), depression (*p* < 0.01), and state anger (*p* < 0.01). Participants who completed the program experienced greater self-esteem and showed reduced scores in terms of depression and state anger. The treatment explained 35%, 24%, and 15% of the variance of these scores, respectively.

*Hypothesis c*. Posttreatment scores were compared with scores obtained from constructed noncausal baseline group (Table 4). Differences decreased for all variables once the intervention program was completed; however, only the depression and trait anger variables showed statistically significant differences with respect to the constructed noncausal baseline group, with a small effect size (15%) for depression and very small one (8%) for trait anxiety.

*Hypothesis d*. The percentage of family members with substance abuse problems who sought out some kind of treatment or intervention service for their problem was found to be 60% (15 out of 25), according to the information gathered during the study period, that is, before treatment program completion.

## 4. Discussion

Although improvement was not observed in all the variables under study as was posited in Hypothesis b, the parents of substance-abusing adolescents/youth who took part in the program did show increased self-esteem and improved mood and anger expression, which is in keeping with previous studies [6, 23, 24]. This improvement in mental health gave rise to scores similar to those observed in the CNCB group (Hypothesis c), which is in line with a similar study in the field [39].

The results obtained in this study suggest that the CRAFT program [35], when applied to families of adolescent/young adult substance users, seems to yield results that are in line with those obtained in research conducted on other non-Spanish populations [6, 23, 24, 37, 42]. Not only does the therapy conducted as part of this program seem to have a beneficial effect on the mental well-being of parents, but it also gives them the tools they need to encourage their children to enter addiction treatment [17] that will help them reduce and/or cease using drugs [15]. In this study, 60% of the substance-abusing adolescents/young adults contacted a treatment program of one kind or another. These results are in keeping with other research in this field that has yielded results ranging between 55% and 86% [6, 11, 37, 38, 40, 42].

A survey of the literature in this field shows that substance abuse among adolescents/youth has a negative impact on the mental health of parents and other family members who live with substance abusers. This gives rise to symptoms such as physical, mental, and social stress. According to the data obtained from this study, these symptoms lead to low self-esteem, depression, stable anxiety, and anger. This makes it difficult to address substance use and leads to a high degree of powerlessness as they do not know how to proceed in such situations [16].

## 5. Conclusions

From this study it was not possible to ascertain if the CRAFT program works by empowering participants, improving mental health, and providing them with the strength they need to effectively deal with their situation using their own resources, in keeping with Orford et al. [4] and Copello et al. [31], if it works because it gives parents new skills (communication, contingency management, and problem solving) which allow them to take control of the family situation [35], or if it involves a combination of both mechanisms. What the study seems to show is that the CRAFT program generates a change in family atmosphere which encourages the adolescent/young adult to enter treatment, improving the mental health of their family members.

This study showed some limitations, which meant that we cannot deem its results to be conclusive. This is mainly due to the study design type, sample size, and the fact that a follow-up of the program at 12 months was not possible. The sample size of the CNCB (similar to that of the experimental group but comprising family members that were not experiencing a stressful family situation) was too reduced to serve as a gauge for a population; for its creation, the study team relied solely upon the information provided to them by the parents themselves. With regard to design, CNCB group is a nonequivalent comparison group. Despite these limitations, the results are encouraging, and further research along these lines would allow us to perfect and build upon this approach in the treatment of families of adolescents/youth with substance abuse problems.

## Figures and Tables

**Table 1:** Sociodemographic variables (in %, except age, in years).

	Experimental group	CNCB group	Children of EG members with AOD use problem
*Sex*			
Male	24	32	98
Female	76	68	2
*Age*			
Mean	51.6	48.2	21.4
Standard deviation	6.8	8.3	4.8
*Marital status*			
Married or domestic partnership	88	84	
Separated or divorced	12	12	
Widow/er	—	4	
*Number of children*			
1	8	4	
2	92	88	
3	—	8	
*Educational level*			
Primary education	84	64	96
Secondary education	8	24	4
Higher education	8	12	—
*Principal drug*			
Cannabis			56
Cocaine			28
Alcohol			12
Heroin			4

**Table 2:** Comparison between experimental group (before treatment) and CNCB group.

Variables	Instrument		Experimental group (before treatment)		CNCB group		*Z*	*p*	TE(*r*)	*r* ^2^
			Mean	SD	Mean	SD				
Emotional variables	Rosenberg	Self-esteem	27.88	4.81	33.56	2.65	5.18	0.000^*∗∗*^	0.65	0.42
	BDI-II	Depression	13.82	10.12	2.60	2.90	−5.07	0.000^*∗∗*^	0.60	0.36
	STAI	S. anxiety	27.04	11.41	24.36	6.95	−1.00	0.478	0.10	0.01
		T. anxiety	27.46	9.78	22.24	6.93	−2.09	0.006^*∗∗*^	0.38	0.14
	STAXI-2	S. anger	24.36	9.97	17.72	3.34	−3.16	0.001^*∗∗*^	0.44	0.19
		T. anger	19.68	5.07	16.04	3.36	−2.99	0.009^*∗∗*^	0.37	0.14
		AEI^1^	29.80	12.26	27.36	8.13	−0.83	0.443	0.11	0.01

^1^Anger expression index.

^*∗∗*^(*p* < 0.01).

**Table 3:** Effect of treatment on study variables in experimental group.

Variables	Instrument		Experimental group				*Z*	*p*	TE(*r*)	*r* ^2^
			Pre		Post					
			Mean	SD	Mean	SD				
Emotional variables	Rosenberg	Self-esteem	27.88	4.81	32.28	4.56	−4.197	0.000^*∗∗*^	0.59	0.35
	BDI-II	Depression	13.82	10.12	8.80	8.52	3.480	0.000^*∗∗*^	0.49	0.24
	STAI	S. anxiety	27.04	11.41	24.20	10.43	1.333	0.188	0.19	0.04
		T. anxiety	27.46	9.78	25.88	11.60	0.444	0.667	0.06	0.00
	SATXI-2	S. anger	24.36	9.97	20.20	8.67	2.764	0.004^*∗∗*^	0.39	0.15
		T. anger	19.68	5.07	19.04	6.34	0.203	0.847	0.03	0.00
		AEI^1^	29.80	12.26	25.64	11.02	1.496	0.139	0.21	0.04

^1^Anger expression index.

^*∗∗*^(*p* < 0.01).

**Table 4:** Comparison between experimental group (after treatment) and CNCB group.

Variables	Instrument		Experimental group (after treatment)		CNCB group		*Z*	*p*	TE(*r*)	*r* ^2^
			Mean	SD	Mean	SD				
Emotional variables	Rosenberg	Self-esteem	32.28	4.56	33.56	2.65	1.31	0.195	0.18	0.03
	BDI-II	Depression	8.80	8.52	2.60	2.90	−2.74	0.006^*∗∗*^	0.39	0.15
	STAI	S. anxiety	24.20	10.43	24.36	6.95	0.29	0.776	0.04	0.00
		T. anxiety	25.88	11.60	22.24	6.93	−1.43	0.156	0.20	0.04
	STAXI-2	S. anger	20.20	8.67	17.72	3.34	−0.58	0.566	0.08	0.01
		T. anger	19.04	6.34	16.04	3.36	−1.99	0.045^*∗*^	0.28	0.08
		AEI^1^	25.64	11.02	27.36	8.13	0.15	0.881	0.02	0.00

^1^Anger expression index.

^*∗*^(*p* < 0.05).

^*∗∗*^(*p* < 0.01).
